# Reconstruction of neglected traumatic Achilles tendon rupture in a young girl

**DOI:** 10.1007/s10195-012-0178-y

**Published:** 2012-01-31

**Authors:** C. Tudisco, S. Bisicchia

**Affiliations:** Department of Orthopaedic Surgery, University of Rome “Tor Vergata”, Viale Oxford 81, 00133 Rome, Italy

**Keywords:** Neglected Achilles tendon rupture, Child, Surgical treatment

## Abstract

Posttraumatic neglected Achilles tendon ruptures in a young patient have not been described in the literature to our knowledge; indeed, neglected ruptures of the Achilles tendon have only rarely been described in adults. We present the case of a 7 year old girl with posttraumatic neglected rupture of the Achilles tendon that was operated on 8 weeks after the trauma.

## Introduction

Achilles tendon rupture is defined as being neglected if the diagnosis is made at least 4 weeks after the trauma [1]. Neglected cases have a tendency to develop complications and show poor functional results. All of the cases described in the literature were adult patients, and they were treated with various surgical techniques using different tissues for augmentation.

The authors report the case of a 7 year old girl with posttraumatic neglected rupture of the Achilles tendon treated 8 weeks after the trauma with a tenorrhaphy using the Bunnell technique augmented with the plantaris gracilis tendon [2].

## Case report

A 7 year old girl was seen at our clinic for pain at the right calf with limping and weakness during plantar flexion of the right foot. Her mother stated that she had started to limp about 8 weeks before after a swinging door hit her on the back of the leg while she was running. At that time she complained of a small cut on the skin that was over her Achilles mid-tendon. She was then taken to the emergency department of the closest hospital, where the skin was sutured. She was allowed to walk but she started to limp on her right lower limb and to feel a kind of weakness during plantar flexion. She was seen again by a pediatrician after 6 weeks, who then referred her to us.

**Fig. 1:**
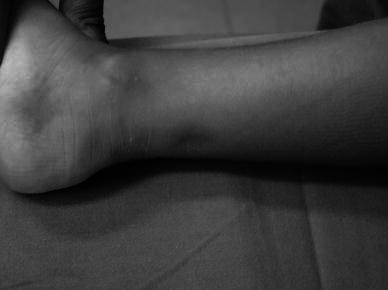
An Achilles tendon gap was present under the skin at physical examination

**Fig. 2:**
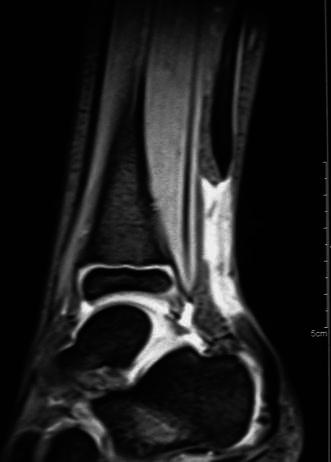
A chronic full-thickness tear of the right Achilles tendon with a gap of 5 cm was present on MRI

Physical examination showed an Achilles tendon gap under the skin at inspection and upon palpation of the area (Fig. 1) and an inability to raise her heel off the ground on the right side. A swollen mass was also present during palpation at the proximal part of the calf muscle, and a positive Thompson squeezing test was noted. Magnetic resonance imaging (MRI) showed a chronic full-thickness tear of the right Achilles tendon with a gap of 5 cm (Fig. 2).

**Fig. 3:**
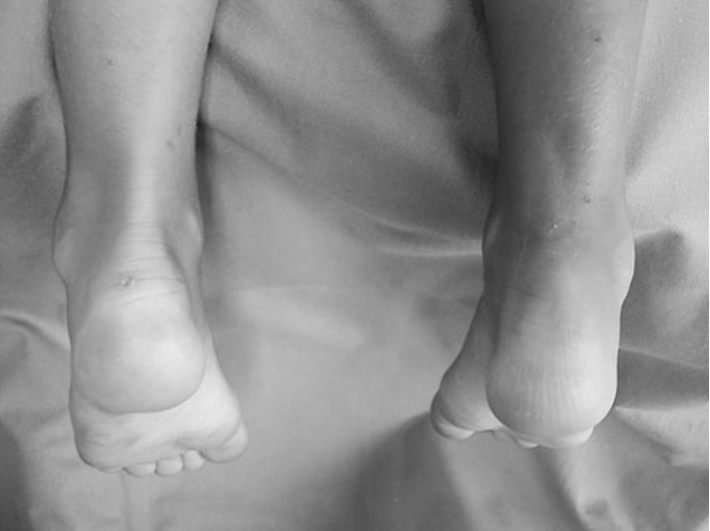
Under general anesthesia and in the prone position, the right foot showed slight dorsiflexion due to the prevalence of the extensor muscles

**Fig. 4:**
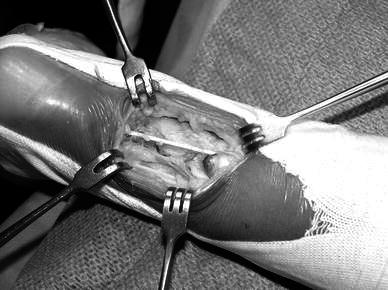
The proximal part of the tendon appeared to be severely proximally retracted, while a small stump of the distal insertion was present over the posterior calcaneal tuberosity. The plantaris gracilis tendon was found to be intact

**Fig. 5:**
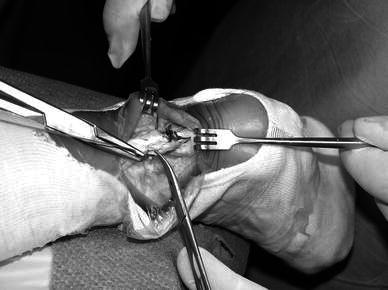
A termino-terminal tenorrhaphy was then possible, but only with the foot flexed in the full equinus position, utilizing the plantaris gracilis tendon as an augmentation (grasped with the clamp)

She was then taken to the operating room. Under general anesthesia and in the prone position, the right foot showed slight dorsiflexion due to the prevalence of the extensor muscles (Fig. 3). After the right lower limb was exanguinated, a medial para-Achilles tendon incision was made just over the gap. The proximal part of the tendon appeared to be severely proximally retracted, while a small stump of the distal insertion was present over the posterior calcaneal tuberosity. The proximal part of the tendon was released from all of the scar tissue and the plantaris gracilis tendon was found to be intact (Fig. 4). A termino-terminal tenorrhaphy was then possible, but only with the foot flexed in the full equinus position, utilizing the plantaris gracilis tendon as an augmentation (Fig. 5). The skin was then closed under extreme tension with reabsorbable sutures. A non-weight-bearing short leg cast in the equinus position was then used for 2 weeks, followed by a new short leg cast in a less equinus position to recover the plantigrade position for three weeks. After the cast was removed, a physical therapy program was prescribed for 3 weeks to recover the correct plantigrade position as well as tip-toed walking. She was able to return to normal activities within a month after cast removal.

The patient was completely asymptomatic at 3 years follow-up.

The parents of the young patient gave informed consent prior to their daughter being included in the study.

## Discussion

Acute traumatic rupture of the Achilles tendon of a child has rarely been described in the literature. More frequently, children are affected by spontaneous avulsion of the Achilles tendon due to the effects on the calcaneus of various conditions such as inflammatory and autoimmune diseases, infectious diseases, or neurological and genetically determined collagen abnormalities [3]. To our knowledge, there are only 2 cases in the literature of a posttraumatic acute rupture at the middle third of the Achilles tendon (the typical localization of an Achilles rupture in an adult) in a young patient. In one case (a 14 year old girl), the tendon, which was initially only partially damaged, was totally ruptured in a subsequent trauma 2 weeks later. She was then operated on, and went on to achieve a full clinical recovery [4]. The second patient, a 7 year old girl, who was the first case of acute Achilles tendon rupture in a child under the age of 10 years to be described in the literature, was successfully treated conservatively for an acute tear of her Achilles tendon after she had fallen on her plantar-flexed foot [5].

Our patient was also a 7 year old patient. She was struck across the mid-tendon sideways by a swinging door, but the lesion at the Achilles tendon was not diagnosed immediately. Neglected traumatic Achilles tendon ruptures in children are not reported in the literature. Nonoperative treatment can be successful only if the tendon is immobilized in the full equinus position. Surgical treatment is often recommended for older and relatively athletic patients, or in cases where treatment has been delayed [3]. In this case, the patient was allowed to walk for 6 weeks after the trauma, even though she was limping due to weakness during plantar flexion. At the operation, we found an large gap between the two edges of the tendon. The proximal one was very retracted and surrounded by scar tissue. Reconstruction using the Bunnell technique [2] was difficult and was only possible in the fully equinus position. Skin closure was also very difficult, with delayed healing of the central part of the scar was present, probably because of ischemia due to the tension at the edges of the skin.

Several different techniques have been described for the treatment of chronic or neglected Achilles tendon ruptures in adults, such as bridging approximation procedures, V–Y slide lengthening of the tendon, gastrocnemius advancement, fascial turndown flaps, local tendon transfers, free tissue transfer, and the use of synthetic grafts [6–8]. The results of all these procedures have only been reported for skeletally mature patients, and their long-term effects in children are unknown. In our young patient, we decided to perform a more anatomical procedure: a termino-terminal tenorrhaphy. We used the plantaris gracilis tendon as an augmentation because it is inconstant and its absence does not influence the function of the ankle.

Complete healing of the scar was obtained after 20 days. A physical therapy program was needed after cast removal to recover the full range of ankle motion. Excellent functional results were observed by 3 months after the surgery. The incision had healed completely and the foot was plantigrade, the patient could walk on tip toes, and she was able to perform 20 repetitive single-leg heel-raise maneuvers. The AOFAS score for the ankle and hindfoot was 96 points (out of a maximum possible score of 100 points), due to moderate restriction of the sagittal motion. At follow-up 3 years after surgery, the patient had recovered full range of motion of the ankle and full ankle function.

To our knowledge, this is the first neglected rupture of the Achilles tendon in a child described in the literature. We believe that in patients under the age of 10 years, nonoperative treatment of acute lesions is safe and offers satisfactory results after a relatively short period of immobilization [5]. In neglected cases, operative treatment is necessary [3], even in the rare case of a child. Selecting the specific surgical procedure to use is a problematic task. Several well-validated techniques are available for adults, but their long-term effects in skeletally immature patients are unknown. Problems with closing the tendon gap and skin closure are always possible for gastrocnemius muscle and skin retraction.
